# Personalized risk prediction for prolonged ileus after minimally invasive colorectal cancer surgery: in-depth risk factor analysis and model development

**DOI:** 10.1007/s00384-024-04693-w

**Published:** 2024-07-23

**Authors:** Wenchao Xiu, Yalin Zhang, Yifan Man, Zongping Yu, Dawei Ren

**Affiliations:** 1 Department of Anorectal Center, Qilu Hospital (Qingdao), Cheeloo College of Medicine, Shandong University, Qingdao, 266035 Shandong China; 2 Department of Breast Surgery, Qingdao Central Hospital, University of Health and Rehabilitation Sciences (Qingdao Central Hospital), Qingdao, 266042 Shandong China; 3 Department of Emergency General Surgery, The Affiliated Hospital of Qingdao University, Qingdao, 266000 Shandong China; 4 Qingdao Women and Children’s Hospital, Qingdao, 266034 China; 5 Department of General Surgery, Qilu Hospital (Qingdao), Cheeloo College of Medicine, Shandong University, Qingdao, 266035 Shandong China

**Keywords:** Colorectal cancer, Minimally invasive, Prolonged postoperative ileus, Risk factors

## Abstract

**Purpose:**

Despite the increasing preference for minimally invasive surgery for colorectal cancer (CRC), the incidence of prolonged postoperative ileus (PPOI) remains high. Thus, this study aimed to identify risk factors for PPOI in patients with CRC who underwent minimally invasive surgery (MICRS) and to develop a practical nomogram for predicting individual PPOI risk.

**Methods:**

A consecutive series of 2368 patients who underwent MICRS between 2013 and 2023 at two tertiary academic centers were retrospectively studied. Using the data from 1895 patients in the training cohort, a multivariable logistic regression model was employed to select significant variables for the construction of a best-fit nomogram. The nomogram was internally and externally validated.

**Results:**

PPOI occurred in 9.5% of patients. Six independent risk factors were identified to construct a nomogram: advanced age (OR 1.055, *P* = 0.002), male sex (OR 2.914, *P* = 0.011), age-adjusted Charlson comorbidity index ≥ 6 (OR 2.643, *P* = 0.025), preoperative sarcopenia (OR 0.857, *P* = 0.02), preoperative prognostic nutritional index (OR 2.206, *P* = 0.047), and intraoperative fluid overload (OR 2.227, *P* = 0.045). The AUCs of the model for predicting PPOI in the training and external validation cohorts were 0.887 and 0.838, respectively. The calibration curves demonstrated excellent consistency between the nomogram-predicted and observed probabilities in both cohorts. Individuals with a total nomogram score of < 197 or ≥ 197 were considered to be at low or high risk for PPOI, respectively.

**Conclusions:**

The integrated nomogram we developed could provide personalized risk prediction of PPOI after MICRS. This quantification enables surgeons to implement personalized prevention strategies, thereby improving patient outcomes.

**Supplementary Information:**

The online version contains supplementary material available at 10.1007/s00384-024-04693-w.

## Introduction

Postoperative ileus (POI), which is generally characterized as a transient nonmechanical disorder of gastrointestinal motility that occurs after abdominal surgery, frequently causes abdominal distension, nausea, and vomiting, as well as intolerance of an oral diet [1]. Its pathophysiology involves an intricate interaction between sympathetic and inflammatory pathways triggered by surgical trauma [2]. Typically, POI resolves within 3 days postoperatively, but may persist or reoccur, which is referred to as prolonged postoperative ileus (PPOI) [3]. A series of recent studies have reported that the prevalence of PPOI in patients with colorectal cancer (CRC) ranges from 7 to 25.9%, with the variation attributable to different definitions [4–7]. PPOIs present a significant healthcare burden, resulting in prolonged hospital stays, increased healthcare costs, and increased morbidity and mortality, necessitating timely identification and intervention [1, 4, 8].

Over the past decade, the management of PPOI has shifted from a “supportive” stance to a more “proactive” strategy with the goal of identifying, preventing, and intervening in the perioperative risk factors that contribute to PPOI. However, accurate and early diagnosis of PPOI remains difficult due to the lack of appropriate laboratory parameters, and there is still a lack of effective therapeutic options for treating PPOI; therefore, it is important to identify patients at high risk of PPOI and intervene early with preventive strategies. Consequently, it becomes imperative, albeit challenging, for surgeons to enhance accuracy in risk stratification and identification of individuals at high risk of PPOI. This facilitates the implementation of evidence-based perioperative interventions to optimize patient care.

Minimally invasive colorectal cancer surgery (MICRS) has been widely recognized for its potential benefits in reducing postoperative complications and enhancing postoperative recovery [9, 10]. Despite the general consensus that MICRS is less invasive, less immunosuppressive, and more conducive to restoring bowel motility than conventional open surgery, the incidence of PPOI has not been reduced significantly to date [10, 11]. In some cases, the benefits conferred by the MICRS and ERAS protocols may even be counterbalanced by PPOI [12]. However, to date, no study has explicitly investigated the risk factors associated with PPOI in patients treated with MICRS.

We hypothesized that identifying risk factors and reliably estimating the probability of an individual experiencing PPOI would facilitate the formulation of targeted prevention and intervention strategies. Therefore, the objective of this study was to identify potential clinical and histopathological risk factors for PPOI in patients who underwent MICRS and to develop an individualized nomogram to predict the probability of PPOI.

## Materials and methods

### Ethics approval and eligibility criteria

**Fig. 1:**
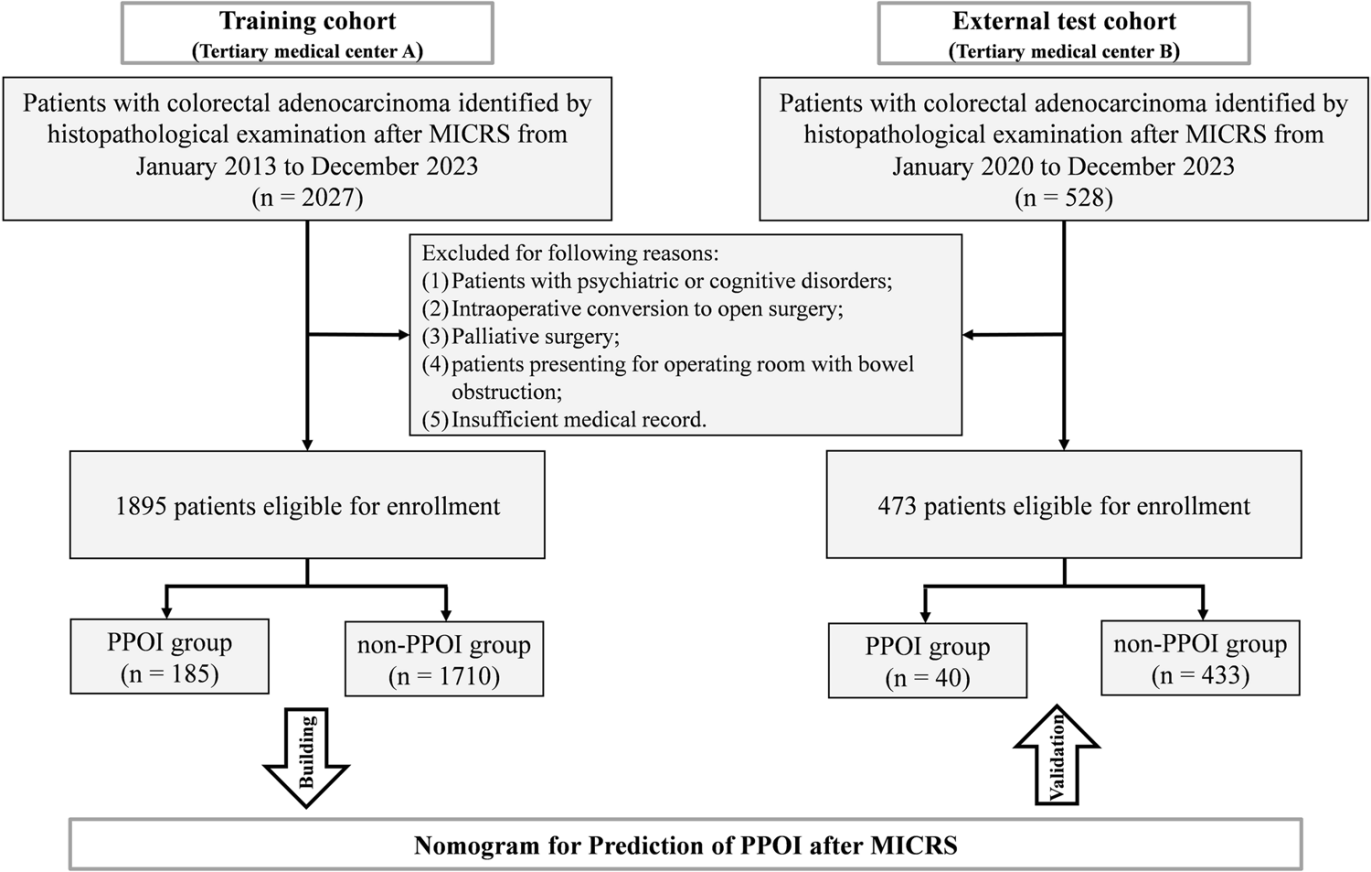
Flowchart of patient enrollment. MICRS, minimally invasive colorectal cancer surgery; PPOI, prolonged postoperative ileus

This was a retrospective cohort study that proposed a predictive model for PPOI. Consecutive patients who underwent MICRS between January 2013 and December 2023 at two tertiary care centers were identified. The data from the Affiliated Hospital of Qingdao University were used as a training cohort to develop a nomogram for predicting PPOI. The predictive model was validated externally using data from Qilu Hospital (Qingdao), Cheeloo College of Medicine, Shandong University. The inclusion criteria were as follows: (1) ambulatory adults (aged ≥ 18 years) who underwent robotic or laparoscopic surgery; (2) histologically confirmed colorectal adenocarcinoma; and (3) preoperative imaging and intraoperative exploration confirming the absence of distant metastases. The exclusion criteria were as follows: (1) patients with psychiatric illnesses such as schizophrenia and patients with cognitive disorders such as dementia; (2) conversion to open surgery; (3) palliative surgery; (4) patients presenting for operating room with bowel obstruction; and (5) insufficient medical records. The detailed flowchart for selecting patients is shown in Fig. 1. This study protocol was reviewed and approved by the ethics committee of each institution, and written informed consent was waived due to its retrospective design. The Transparent Reporting of a Multivariable Prediction Model for Individual Prognosis or Diagnosis (TRIPOD) guidelines were followed to ensure the quality of data reporting [13].

### Definition of postoperative prolonged ileus

The diagnostic criteria for PPOI were informed by definitions extracted from 52 trials identified in a global survey and systematic review [6]. Specifically, PPOI is defined as the presence of at least two of the following criteria on or after postoperative day (POD) 4:

1. Moderate to severe nausea or vomiting experienced within the last 12 h;
2. Inability to tolerate semi-solid or solid diets within the past 24 h;
3. Lack of flatus and defecation within the past 24 h;
4. Moderate to severe abdominal distension;
5. Radiological (CT or X-ray) findings, including stomach distension, air-fluid planes, or dilated bowel without transition points.

### Observable variables/predictors

Thirty-seven key variables were extracted for the potential risk factor analysis of PPOI. The baseline characteristics included age, gender, body mass index (BMI), comorbidities, ASA scale, age-adjusted Charlson Comorbidity Index (CCI), sarcopenia, smoking, alcohol consumption, preoperative serologic indicators, previous abdominal surgery, and neoadjuvant therapy. Preoperative serologic indicators were selected from the most recent laboratory results prior to surgery. The prognostic nutritional index (PNI) was calculated using the following formula: 5 × total lymphocyte count (10^9^/L) + serum albumin (g/L) [14]. Two skilled researchers employed the semiautomatic outlining feature of Infinitt PACS software to delineate the skeletal muscle area (SMA) at the level of the third lumbar vertebra on preoperative CT cross-sectional images [15]. Thus, the skeletal muscle index (SMI) was determined using the following formula: SMA (cm^2^)/height (m)^2^. Sarcopenia was identified based on established sex-specific thresholds: SMI < 34.9 cm^2^/m^2^ for women and < 40.8 cm^2^/m^2^ for men, as validated in previous studies [16].

Operative characteristics encompassed tumor site, surgical approach (laparoscopic or robotic), estimated blood loss, stoma formation, operative time, specimen extraction techniques, intraoperative fluid overload, and pathological findings. As reported in previous studies, fluid supplementation ≥ 3 mL/kg/h was considered the reference value for the intraoperative fluid overload threshold [17, 18].

The postoperative recovery indices included postoperative pain, the duration of abdominal drain indwelling, the time to nasogastric tube insertion, length of hospital stay, complications, and surgical mortality. Postoperative pain intensity was measured using the visual analog scale (VAS). Postoperative complications were defined as any adverse events occurring within 30 days after surgery. All-cause mortality within 30 days of surgery was considered surgical mortality.

### Types of interventions

At our center, patients with suspected or confirmed PPOI were generally treated with the following combination of interventions, partially adjusted to the patient’s condition and the attending surgeons: (1) fasting and gastrointestinal decompression; (2) sham feeding: gum chewing (30 min several times a day until flatus); (3) correction of acidaemia, restoration of electrolyte concentration and provision of nutritional support; (4) enhancement of early ambulation; (5) removal of abdominal drainage tubes and urinary catheters as soon as possible; (6) acupuncture: the Zu-san-li, Shang-ju-xu, and San-yin-jiao acupoints on both lower extremities were identified to deliver traditional acupuncture or transcutaneous acupoint electrical stimulation (TEAS). Adequate current stimulation at a frequency of 2–10 Hz (TEAS instrument: SDZ-V; Hwato, China) was indicated when effective tingling sensations occurred at these points. The TEAS was then adjusted to the maximum intensity that the patient could tolerate and was sustained for 30 min. (7) Prokinetic agents (e.g., domperidone, metoclopramide, mosapride, or erythromycin); (8) multimodal analgesia to optimize analgesia and minimize opioid-related adverse effects; and (9) psychological interventions to eliminate anxiety and emotional burden.

### Nomogram construction and evaluation

Data analysis was conducted using R Project software (version 4.2.3, http://www.R-project.org). In the training cohort, univariable logistic regression analysis was used to analyze the correlation between each variable and PPOI. Variables with *P* < 0.05 in the univariable analysis were further included in the multivariable logistic regression analysis. A predictive nomogram model was then constructed based on independent variables (*P* < 0.05) identified by the multivariable logistic regression analysis. The optimal cutoff value of the nomogram was obtained from the maximum Youden index. The Youden Index is a commonly used method in diagnostic test evaluation, defined as *J* = Sensitivity + Specificity − 1. By maximizing this index, we identified the threshold that provides the best balance between sensitivity and specificity for predicting the outcome of interest.

In addition, the nomogram was internally and externally validated. The discrimination of the model was evaluated by the area under the receiver operating characteristic curve (AUC). Calibration curves were plotted using 1000 bootstrap resamples to assess the consistency between the nomogram-predicted probability and observed probability, and the 45-degree line was used as a perfect model with 100% accuracy. Finally, the clinical applicability of the nomogram was assessed by decision curve analysis (DCA) and clinical impact curve (CIC). A *P* value less than 0.05 was considered statistically significant.

## Results

### Eligible patients

A total of 2368 eligible patients were enrolled, which included 1895 patients as the training cohort and 473 patients as the external validation cohort. Among the cohorts, 1982 (83.7%) patients were treated with laparoscopic CRC surgery, while 386 (16.3%) underwent robotic CRC surgery. Baseline characteristics demonstrated equilibrium between the training cohort and the external test cohort (Table 1). The median (interquartile range, IQR) age of all patients was 67 (58–72) years, with a male predominance (1439/2368, 60.7%).

**Table 1:** Patient, laboratory, operative, and tumor characteristics in the training and external test cohorts

Variable^a^	Overall (*n* = 2368)	Training cohort (*n* = 1895)	External test cohort (*n* = 473)	*P* value
PPOI, *n* (%)				0.386
Yes	225 (9.5)	185 (9.8)	40 (8.5)	
No	2143 (90.5)	1710 (90.2)	433 (91.5)	
Age, median (IQR), y	65 [58, 72]	65 [56, 71]	67 [58.5, 74]	0.484
Sex, *n* (%)				0.321
Male	1439 (60.7)	1161 (61.3)	278 (58.8)	
Female	929 (39.2)	734 (38.7)	195 (41.2)	
Body mass index, kg/m^2^, median (IQR)^b^	23.8 [20.8, 25.6]	24.1 [21.7, 25.8]	23.3 [20.4, 24.7]	0.337
American Society of Anesthesiologists score, n (%)				0.097
1 or 2	2086 (88.0)	1679 (88.6)	406 (85.8)	
3 or 4	283 (12.0)	216 (11.4)	67 (14.2)	
Comorbidity, *n* (%)				0.08
Yes	1302 (54.8)	1025 (54.1)	277 (58.6)	
No	1066 (45.2)	870 (45.9)	196 (41.4)	
Age-adjusted Charlson comorbidity Index score, median (IQR)				0.216
< 6	1958 (82.7)	1576 (83.2)	382 (80.8)	
≥ 6	410 (17.3)	319 (16.8)	91 (19.2)	
Preoperative sarcopenia				0.172
Yes	2106 (88.9)	1677 (88.5)	429 (90.7)	
No	262 (11.1)	218 (11.5)	44 (9.3)	
Smoking, *n* (%)				0.444
No	1745 (73.7)	1403 (74)	342 (72.3)	
Yes	623 (26.3)	492 (26)	131 (27.7)	
Alcohol consumption, *n* (%)				0.147
No	1443 (60.9)	1141 (60.2)	302 (63.8)	
Yes	925 (39.1)	754 (39.8)	171 (36.2)	
Neoadjuvant therapy, *n* (%)				0.661
No	1980 (83.6)	1576 (83.2)	404 (85.4)	
Yes	388 (16.4)	319 (16.8)	69 (14.6)	
Previous abdominal surgery, *n* (%)				0.265
No	2135 (90.2)	1715 (90.5)	420 (88.8)	
Yes	233 (9.8)	180 (9.5)	53 (11.2)	
Preoperative nutritional and inflammatory statuses, median (IQR)				
C-reaction protein, mg/L	2.35 [1.59, 3.69]	3.09 [1.29, 5.31]	2.15 [1.59, 2.56]	0.341
Serum albumin level, g/L	39.5 [37.7, 43.3]	38.9 [34.3, 43]	40.65 [37.95, 43.97]	0.27
Serum potassium level, mmol/L	4.18 [3.79, 4.54]	4.24 [3.96, 4.32]	3.92 [3.77, 4.62]	0.408
Serum sodium level, mmol/L	141 [140, 142]	141 [137.5, 142]	141 [140, 142]	0.877
Serum leukocyte count, 10^9^/L	5.79 [4.43, 7.53]	5.79 [5.2, 7.22]	5.69 [4.14, 8.30]	0.951
Serum neutrophil count, 10^9^/L	3.85 [3.06, 5.05]	4.02 [3.31, 4.98]	3.42 [2.84, 6.18]	0.646
Serum lymphocyte count, 10^9^/L	1.43 [1.10, 1.85]	1.42 [1.22, 1.55]	1.56 [1.05, 1.9]	0.759
Serum monocyte count, 10^9^/L	0.37 [0.27, 0.48]	0.36 [0.27, 0.51]	0.38 [0.27, 0.44]	0.668
Hemoglobin level, g/L	112 [96, 135]	98 [66, 135]	113.5 [102.5, 139.5]	0.098
Platelet count, 10^9^/L	226 [157, 307]	242 [149, 308]	209 [163.75, 248.5]	0.51
NLR	3.14 [2.23, 4.07]	2.78 [2.13, 4.32]	3.30 [2.42, 4.00]	0.976
LMR	3.72 [2.64, 5.11]	3.72 [2.64, 5.04]	3.76 [2.68, 5.14]	0.783
PLR	158.2[98.5, 214.5]	174.1 [88.8, 266.7]	153.1 [105.4, 186.6]	0.462
PNI	48.2 [42.3, 51]	47.1 [41.6, 51]	49.2 [43.4, 51.5]	0.701
Tumor site, *n* (%)				0.15
Colonic	1056 (44.6)	859 (45.3)	197 (41.6)	
Rectal	1312 (55.4)	1036 (54.7)	276 (58.4)	
Tumor size, cm, median (IQR)	4.5 [3.5, 6]	4.5 [3.35, 6.0]	5.0 [3.62, 6.25]	0.116
Pathological TNM stage, *n* (%)^c^				0.494
I	128 (5.4)	105 (5.5)	23 (4.9)	
II	1472 (62.2)	1167 (61.6)	305 (64.5)	
III	768 (32.4)	623 (32.9)	145 (30.7)	
Surgical approach				0.337
Robotic	386 (16.3)	302 (15.9)	84 (17.8)	
Laparoscopic	1982 (83.7)	1593 (84.1)	389 (82.2)	
Type of operation				0.067
Right hemicolectomy	491 (20.7)	407 (21.5)	84 (17.8)	
Transverse colectomy	100 (4.2)	73 (3.9)	27 (5.7)	
Left hemicolectomy	465 (19.6)	379 (50.0)	86 (18.2)	
Rectal resection	1312 (55.4)	1036 (54.7)	276 (58.4)	
Intraoperative fluid overload, *n* (%)				0.177
No	1382 (58.4)	1093 (57.7)	289 (61.1)	
Yes	986 (41.6)	802 (42.3)	184 (38.9)	
Specimen extraction techniques, *n* (%)				0.091
Conventional extraction	2348 (99.2)	1882 (99.3)	466 (98.5)	
Natural orifice extraction	20 (0.8)	13 (0.7)	7 (1.5)	
Stoma formation, *n* (%)				0.243
No	2106 (88.9)	1727 (91.1)	439 (92.8)	
Yes	262 (11.1)	168 (8.9)	34 (7.2)	
Estimated blood loss, mL, median (IQR)	75 [50, 85]	75 [50, 82.5]	80 [57.5, 90]	0.164
Operative time, min, median (IQR)	165 [135, 185]	162.5 [130, 178.75]	165 [135, 192.5]	0.36
Perioperative transfusion, *n* (%)				0.517
No	2242 (94.7)	1797 (94.8)	445 (94.1)	
Yes	126 (5.3)	98 (5.2)	28 (5.9)	

Abbreviation: *IQR*, interquartile range; *LMR*, lymphocyte-monocyte ratio; *NLR*, neutrophil–lymphocyte ratio; *PNI*, prognostic nutrition index; *PLR*, platelet-lymphocyte ratio; *PPOI*, prolonged postoperative ileus

^a^Data are presented as median with interquartile range (IQR) if the variables are continuous and not normally distributed, and as frequency (percentage) if the variables are categorical. *P*-values were calculated by the Mann–Whitney test for continuous variables or Pearsons *χ*^2^ test or Fishers exact test for categorical variables

^b^Calculated as weight in kilograms divided by height in meters squared

^c^Staging was performed according to the AJCC Cancer Staging

### Incidence of PPOI and postoperative recovery after MICRS

The incidence of PPOI in the training cohort was 9.8% (Table 1). The results related to postoperative recovery are shown in Table 2. No significant differences in median (IQR) visual analog scale (VAS) scores were found between the PPOI and non-PPOI groups on POD1 [3(2, 4) vs 2(2, 4), *P* = 0.328], POD2 [3(2, 4) vs 3(2, 4), *P* = 0.426], and POD3 [2(1, 3) vs 2(1, 3), *P* = 0.115]. In the PPOI group, postoperative insertion of the nasogastric tube was POD 4.5 (IQR 2–6) and removed 4 (IQR 3–8) days later. There was no significant difference between the two groups in terms of the duration of abdominal drain placement [median (IQR): 4 (3–8.5) vs 4.5 (3–11) days, *P* = 0.072]. Postoperative complications were observed in 32 (17.3%) patients in the PPOI group versus 221 (12.9%) patients in the non-PPOI group (*P* = 0.097). Moreover, data showed no significant difference between the two groups with respect to all-cause mortality in the hospital (2.2% vs 0.8%, *P* = 0.074). However, compared with the non-PPOI group, patients in the PPOI group had a significantly longer median length of hospital stay (12.5 [8–19] vs. 9 [7–13] days, *P* < 0.001).

**Table 2:** Univariate and multivariate logistic regression analyses for risk factors of PPOI following MICRS

Variables	Univariate analysis		Multivariate analysis	
	Crude *OR* (95% *CI*)	*P* value	Adj *OR* (95% *CI*)	*P* value
Age	1.068 (1.041, 1.097)	< 0.001	1.055 (1.021, 1.090)	0.002
Male	2.355 (1.369, 4.052)	0.002	2.914 (1.281, 6.627)	0.011
American Society of Anesthesiologists score ≥ 3	1.358 (0.407, 4.539)	0.619	-	-
Body mass index	1.188 (0.716, 1.970)	0.504	-	-
Comorbidity	1.219 (0.988, 1.505)	0.065	-	-
Diabetes mellitus	2.094 (1.294, 3.388)	0.003	0.620 (0.270, 1.426)	0.261
Age-adjusted Charlson Comorbidity Index score ≥ 6	2.484 (1.532, 4.026)	< 0.001	2.643 (1.351, 5.172)	0.025
Smoking	1.096 (0.313, 3.840)	0.886	-	-
Preoperative sarcopenia	0.861 (0.824, 0.899)	< 0.001	0.857 (0.812, 0.906)	0.02
Alcohol consumption	0.772 (0.238, 2.497)	0.665	-	-
Neoadjuvant therapy	2.659 (0.612, 11.558)	0.192	-	-
Previous abdominal surgery	2.009 (0.992, 4.068)	0.053	-	-
Preoperative nutritional and inflammatory statuses				
C-reaction protein	0.926 (0.789, 1.086)	0.344	-	-
Serum albumin level	2.095 (1.033, 4.247)	0.04	1.690 (0.474, 6.033)	0.419
Serum potassium level	0.393 (0.121, 1.273)	0.119	-	-
Serum sodium level	0.952 (0.780, 1.161)	0.625	-	-
Serum leukocyte count	1.015 (0.856, 1.203)	0.865	-	-
Serum neutrophil count	0.996 (0.802, 1.238)	0.974	-	-
Serum lymphocyte count	1.365 (0.431, 4.329)	0.597	-	-
Serum monocyte count	1.500 (0.334, 6.726)	0.596	-	-
Hemoglobin level	1.020 (0.996, 1.044)	0.107	-	-
Platelet count	0.999 (0.994, 1.005)	0.839	-	-
NLR	1.007 (0.725, 1.399)	0.967	-	-
LMR	0.921 (0.643, 1.321)	0.656	-	-
PLR	1.002 (0.996, 1.008)	0.486	-	-
PNI	2.303 (1.327, 3.998)	0.003	2.206 (1.009, 4.822)	0.047
Tumor site	0.976 (0.503, 1.892)	0.942	-	-
Tumor size	0.838 (0.493, 1.424)	0.514	-	-
Pathological TNM stage	0.592 (0.267, 1.312)	0.197	-	-
Surgical approach	0.963 (0.887, 1.045)	0.364	-	-
Type of operation	0.776 (0.449, 1.343)	0.365	-	-
Stoma formation	0.492 (0.157, 1.172)	0.257	-	-
Intraoperative fluid overload	1.895 (1.095, 3.277)	0.022	2.227 (1.019, 4.870)	0.045
Specimen extraction techniques	2.246 (0.612, 8.250)	0.223	-	-
Operative time	1.955 (1.106, 3.459)	0.021	1.400 (0.592, 3.309)	0.443
Estimated blood loss	1.026 (0.114, 9.226)	0.982	-	-
Perioperative transfusion	2.128 (0.206, 22.038)	0.527	-	-

Abbreviation: *CI*, confidence interval; *LMR*, lymphocyte-monocyte ratio; *MICRS*, minimally invasive colorectal cancer surgery; *NLR*, neutrophil–lymphocyte ratio; *OR*, odds ratio; *PNI*, prognostic nutrition index; *PLR*, platelet-lymphocyte ratio; *PPOI*, prolonged postoperative ileus

### Independent risk factors for PPOI

Univariable and multivariable logistic regression analyses revealed that advanced age (*OR* [95% *CI*], 1.055 [1.021, 1.090]; *P* = 0.002), male sex (*OR* [95% *CI*], 2.914 [1.281, 6.627]; *P* = 0.011), an age-adjusted CCI ≥ 6 (*OR* [95% *CI*], 2.643 [1.351, 5.172]; *P* = 0.025), preoperative PNI (*OR* [95% *CI*], 2.206 [1.009, 4.822]; *P* = 0.047), preoperative sarcopenia (*OR* [95% *CI*], 0.857 [0.812, 0.906]; *P* = 0.02), and intraoperative fluid overload (*OR* [95% *CI*], 2.227 [1.019, 4.870]; *P* = 0.045) were identified as independent risk factors for PPOI (Table 3). Although significant in Univariable analysis, diabetes mellitus, preoperative hypoalbuminemia, and operative time did not remain significant in the multivariable analysis.

**Table 3:** Postoperative recovery in patients with or without PPOI in the training cohort

Variable^a^	Patients with PPOI (*n* = 185)	Patients without PPOI (*n* = 1710)	*P*-value
Mobilization (POD), *n* (%)			0.649
Day 0/1	143 (77.3)	1296 (75.8)	
Day 2 or later	42 (22.7)	414 (24.2)	
Pain—VAS scale, median (IQR)^b^			
POD 1	3 (2, 4)	2 (2, 4)	0.328
POD 2	3 (2, 4)	3 (2, 4)	0.426
POD 3	2 (1, 3)	2 (1, 3)	0.115
Removal of abdominal drains (POD), d, median (IQR)	4 (3, 8.5)	4.5 (3, 11)	0.072
Complications, *n* (%)			0.097
Yes	32 (17.3)	221 (12.9)	
No	153 (82.7)	1489 (87.1)	
Blood transfusion, *n* (%)			0.248
Yes	10 (5.4)	63 (3.7)	
No	175 (94.6)	1647 (96.3)	
Length of hospital stay, d, median (IQR)	12.5 (8–19)	9 (7–13)	< 0.001
In-hospital mortality (yes), *n* (%)			0.074
Yes	4 (2.2)	14 (0.8)	
No	181 (97.8)	1692 (99.2)	

Abbreviations: *IQR*, interquartile range; *POD*, postoperative day; *PPOI*, prolonged postoperative ileus; *VAS*, visual analog scale

^a^Data are presented as median with interquartile range (IQR) if the variables are continuous and not normally distributed, and as frequency (percentage) if the variables are categorical. *P*-values were calculated by the Mann–Whitney test for continuous variables or Pearsons *χ*^2^ test or Fishers exact test for categorical variables

^b^Data on relevant variables were missing for very few participants, and all missing values were filled in with the corresponding median values in each group

### Development and validation of a PPOI-predicting nomogram

**Fig. 2:**
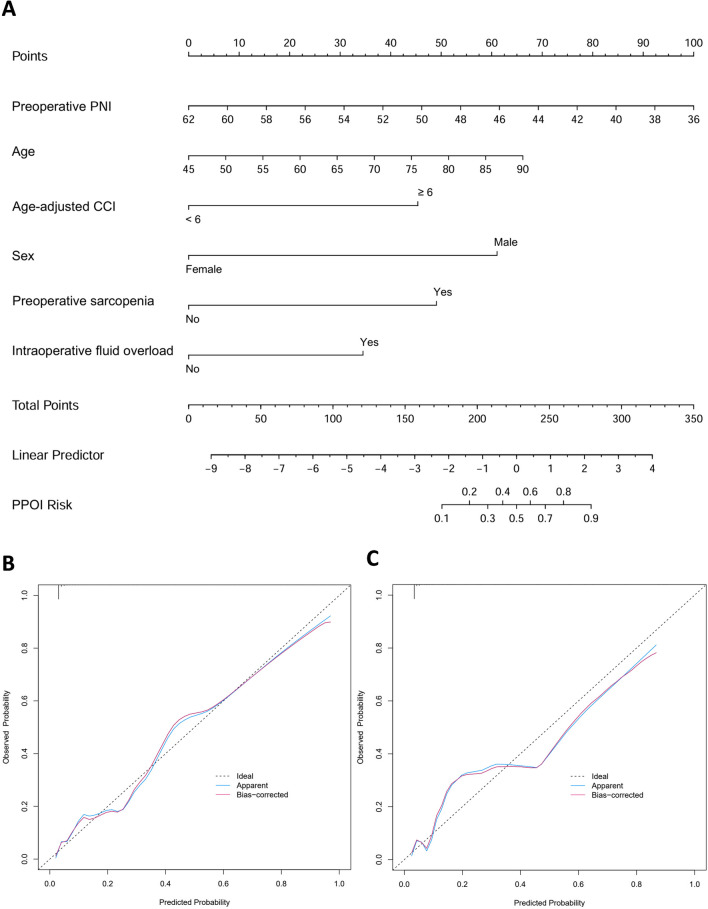
Nomogram developed based on multivariable logistic regression for predicting the probability of PPOI after MICRS (**A**). Calibration plot comparing the predicted and actual probabilities of PPOI in the training cohort (**B**) and external validation cohort (**C**). CCI, Charlson Comorbidity Index; MICRS, minimally invasive colorectal cancer surgery; PNI, prognostic nutrition index; PPOI, prolonged postoperative ileus

The nomogram was constructed based on the weight of six significant predictors in the multivariable logistic regression analysis (Fig. 2A). This nomogram allows the estimation of the risk of PPOI in each patient. Based on the sum of the assigned points for each selected predictor in the nomogram, the larger value of total points indicated a higher risk of PPOI in patients who underwent MICRS. For example, a 70-year-old male patient with an age-adjusted CCI of 6 and a preoperative PNI of 42 who is scheduled to undergo MICRS has a total score of 224 and a corresponding risk of PPOI of 52%. To further stratify the risk of PPOI, we applied the optimal cutoff function and the Youden index to calculate the binary risk stratification threshold of the prediction model. Individuals with a total score of < 197 or ≥ 197 were considered to be at low or high risk for PPOI (Table S1).

First, the validation was performed internally in the training cohort. The AUCs of internal validation were 0.887 (95% CI = 0.837–0.931) (Fig. S1). Furthermore, a comparison of AUC by DeLong’s test revealed that the nomogram had greater predictive power than all of the single independent risk factors (All *P* values > 0.05, Table S2). In the external validation cohort, 473 patients were eligible for analysis using the same eligible criteria and study period, with PPOI incidence of 8.5%. The AUC of external validation were 0.838 (95% CI = 0.738–0.911) (Fig. S1). Calibration curves for internal and external validation were plotted, showing good agreement between the observed and predicted probabilities in both the training and external validation cohorts (Fig. 2B and C).

### Clinical usefulness

**Fig. 3:**
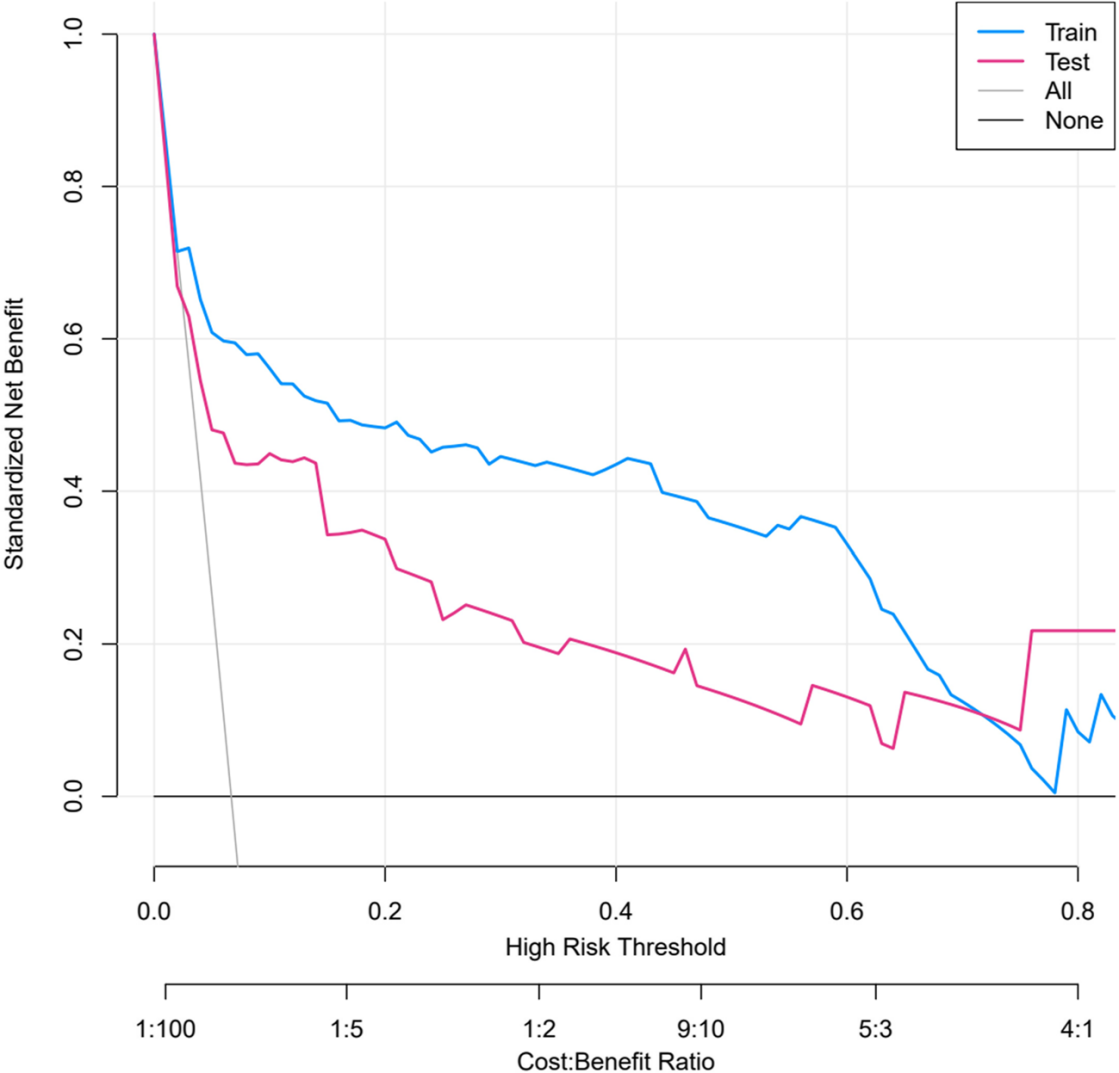
DCA of the nomogram. When the threshold probability was greater than 0.1, using the nomogram to predict PPOI after MICRS had greater net benefit than all-or-none treatment strategies. MICRS, minimally invasive colorectal cancer surgery; PPOI, prolonged postoperative ileus

**Fig. 4:**
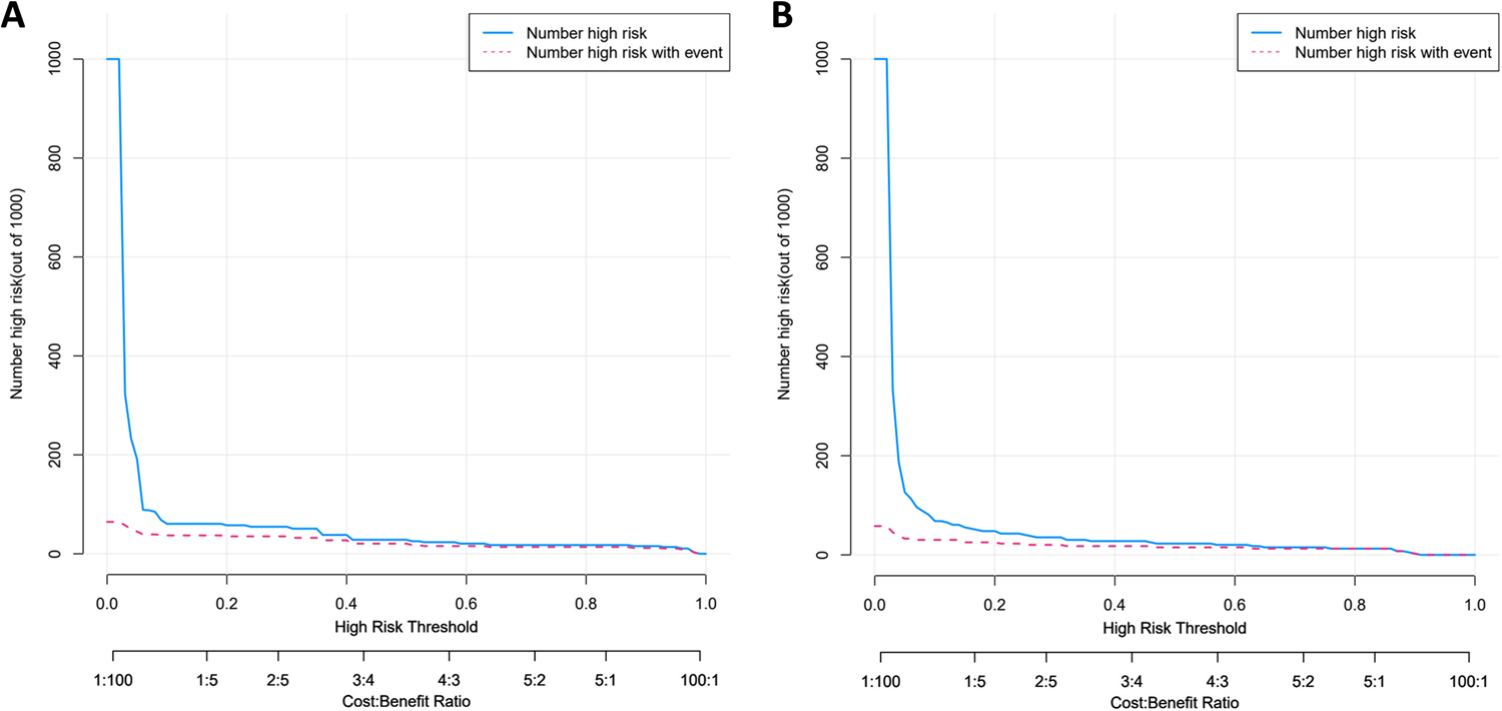
CIC analysis of the nomogram in the training cohort (**A**) and external validation cohort (**B**)

The DCA curves showed good net benefits in both the training cohort and external validation cohort, which indicated the superior diagnostic accuracy of the nomogram, as indicated in Fig. 3. Moreover, CIC confirmed the clinical value of the nomogram by visually indicating that it provided a high clinical net benefit (Fig. 4). Taken together, DCA and CIC demonstrated the clinical applicability of the model.

## Discussion

In this study, thirty-seven potential predictors of PPOI in 2368 patients who underwent MICRS were comprehensively analyzed. Six factors were identified as significantly associated with the occurrence of PPOI: advanced age, male sex, PNI, an age-adjusted CCI ≥ 6, sarcopenia, and intraoperative fluid overload. For visualization, a nomogram was constructed by incorporating the above significant factors. The effectiveness of the nomogram was validated both internally and externally by AUC, calibration curves, DCA, and CIC, confirming the predictive accuracy and clinical utility of the model. Moreover, the main strengths of this study were its recency, comprehensiveness, and generalizability.

The incidence of PPOI in this study was 9.5%, which appears to be lower than the rates reported by Wolthuis et al. (15.9%) [4] and Chapuis et al. (14%) [5], and is similar to those reported by Stamos et al. (12.7%) [19] and Andrea et al. (7%) [20]. One potential reason for this variance could be the lack of a consensus definition for PPOI. The controversy focused mainly on the duration of POI, which should be considered prolonged. For instance, we defined the duration as more than 4 days with reference to the Chinese consensus, while Chapuis et al. [5] specified 3 days, and Svatek et al. [21] specified 6 days. Therefore, it is imperative to formulate a uniform definition of PPOI. Another reason may be that all patients enrolled received MICRS and the widespread implementation of the ERAS program at our center.

Aging is well known to affect gastrointestinal functions [22]. Notably, this study is the first to identify age-adjusted CCI as an independent predictor of PPOI. In prior studies, age-adjusted CCI has been associated with overall survival, disease-specific survival, or treatment-related complications, confirming its predictive validity [23–25]. In addition, population aging is an inevitable consequence of demographic transition and poses a major challenge to contemporary healthcare [26]. This phenomenon indicates that an increasing number of elderly patients will undergo surgery for CRC, potentially increasing the incidence of PPOI. Therefore, it is essential to identify individuals at risk for PPOI and allow early intervention with preventive strategies.

Similarly, the PNI is a newly identified predictor of PPOI risk and has previously been used to assess postoperative nutritional status and prognosis in cancer patients [27, 28]. A lower PNI indicates a higher risk of malnutrition, and it tends to decrease with advancing age [29]. Malnutrition may compromise gut barrier integrity, leading to microbial dysbiosis, altered gut function, and a leaky gut barrier. Thus, timely nutritional intervention for patients identified as malnourished or at nutritional risk is expected to improve their postoperative nutritional status, promote gastrointestinal function recovery, and reduce complications [30]. In addition, the PNI was a stronger predictor of PPOI than its individual components (i.e., serum albumin and total lymphocyte count), particularly in patients aged > 65 years [31].

Preoperative sarcopenia has also been proven to be an independent predictor of PPOI. Based on the findings of Rinaldi et al. [32], we hypothesized that patients with sarcopenia are susceptible to PPOI. Previous studies have shown that sarcopenia is associated with decreases in nutritional indicators such as PNI, hemoglobin, prealbumin, and albumin [33]. In addition, nutritional imbalance has been reported to be associated with a pro-inflammatory state, including elevated levels of chemokines and cytokines [32]. An imbalance between pro-inflammatory and anti-inflammatory cytokines is essential for the pathogenesis of PPOI [34]. Moreover, intestinal smooth muscle contractility is impaired due to collagen accumulation around the enteric plexus [35]. Further basic research is needed to determine the underlying mechanisms.

Optimizing perioperative fluid management may help reduce the risk of PPOI. Shim et al. [36] highlighted a correlation between increased intravenous fluids, PPOI, and prolonged hospital stays. Conversely, Gómez-Izquierdo et al. [37] reported a consistent 22% incidence of POIs across both fluid restriction and control groups, indicating ongoing debate. Similarly, our findings revealed that intraoperative fluid overload was significantly associated with PPOI. This association underscores the potential adverse effects of electrolyte imbalance and tissue edema that often accompany hypervolemia or hypovolemia [38]. As recently discussed, hemodynamic monitoring using devices such as PiCCO and transesophageal Doppler may be helpful in guiding perioperative fluid therapy, but this argument still needs further investigation [39]. Maintaining a zero fluid balance aims to mitigate intestinal edema and prevent PPOI. Hence, optimizing perioperative fluid management and individualizing fluid therapy may help effectively combat PPOI.

We developed a novel nomogram, an efficient and user-friendly graphical tool for predicting the individual probability of PPOI. In addition, we compared its performance and features with existing nomograms in the literature. For instance, Guo et al. [40] included variables such as advantage age, hypoalbuminemia, high surgical difficulty, and postoperative use of opioid analgesic, while Kotaro et al. [41], Fan et al. [42] included variables like gender, smoking, neutrophil–lymphocyte ratio, and open surgery. In contrast, our nomogram was developed for a specific population undergoing minimally invasive surgery and included several independent risk factors, such as age-adjusted CCI, preoperative PNI and sarcopenia, which better reflect nutritional status, and intraoperative fluid overload. All of these variables have been validated for the first time. Moreover, our approach used a larger sample size and more comprehensive multivariable logistic regression analysis. Similarly, we found that age and gender still play a crucial role in the development of PPOI. Additionally, we validated our nomogram both internally and externally, providing a robust assessment of its predictive accuracy. Compared to previous studies, our model performed well in terms of AUC, calibration curve, DCA, and CIC during external validation, making it suitable for patients across different centers. In summary, our nomogram offers several advantages over existing models, including higher predictive accuracy and the inclusion of unique clinical variables. These differences highlight the potential for improved patient outcomes through more targeted interventions. Precision medicine is an emerging aspect of medicine, with individuality and personalization being core treatment principles. Furthermore, the integration of a decision support system based on this model is expected to be more effective in identifying high-risk patients for prophylactic or therapeutic clinical trials.

The present study has several limitations. First, the retrospective study design limited the ability to control for unmeasured confounders or to assess the stability of causality. Although recruitment at a single center may present an inherent limitation, it may indeed reduce potential bias associated with possible differences between surgical attitudes and hospital-based practices. Second, discrepancies in definitions underscore the need for the international scientific community to develop a unified and standardized definition in future investigations. Such standardization would facilitate robust comparisons of outcomes and enhance the reliability of identified risk factors.

## Conclusion

By integrating six independent risk factors, we established an easy-to-use nomogram that accurately predicts the probability of PPOI after MICRS, demonstrating favorable accuracy in both internal and external validation cohorts. Personalized and timely risk assessment allows for more accurate decision-making regarding treatment strategies and the allocation of healthcare resources. Additional data from different centers are needed to validate the robustness and generalizability of the predictive model.

## Supplementary Information

Below is the link to the electronic supplementary material.

Supplementary file1 (DOCX 40 KB)

Supplementary file2 (DOCX 16 KB)

Supplementary file3 (DOCX 19 KB)

Supplementary file4 (PDF 1190 KB)

## Data Availability

The datasets used and/or analyzed during the current study are available from the corresponding author upon reasonable request.
