# Supplementary material for: Personalized risk prediction for prolonged ileus after minimally invasive colorectal cancer surgery: in-depth risk factor analysis and model development
Source: Int J Colorectal Dis. 2024 Jul 23;39(1):115. doi: 10.1007/s00384-024-04693-w (PMC11266276; doi:10.1007/s00384-024-04693-w)
Supplement: Supplementary file 1 — Supplementary file1 (DOCX 40 KB) [file 384_2024_4693_MOESM1_ESM.docx]

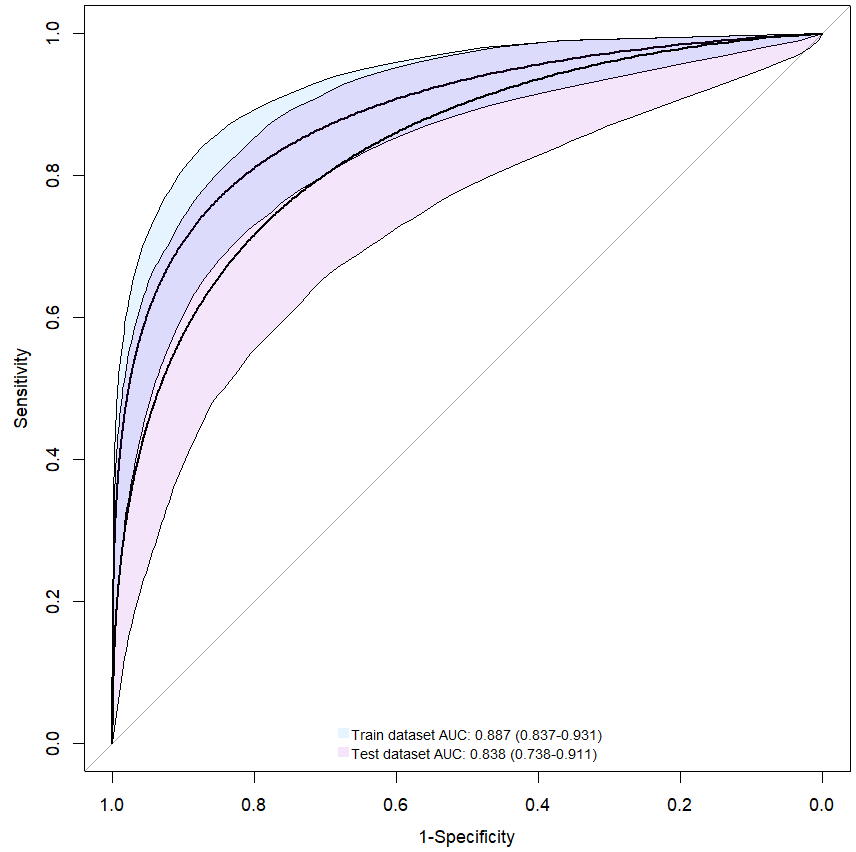


**Figure 3.** AUCs of the nomogram used to predict PPOI following MICRS in the training and external test cohorts. AUC, the area under the receiver operator characteristic curve; MICRS, minimally invasive colorectal cancer surgery; PPOI, prolonged postoperative ileus.
