# Supplementary material for: Personalized risk prediction for prolonged ileus after minimally invasive colorectal cancer surgery: in-depth risk factor analysis and model development
Source: Int J Colorectal Dis. 2024 Jul 23;39(1):115. doi: 10.1007/s00384-024-04693-w (PMC11266276; doi:10.1007/s00384-024-04693-w)
Supplement: Supplementary file 2 — Supplementary file2 (DOCX 16 KB) [file 384_2024_4693_MOESM2_ESM.docx]

**Table S1**. **Optimal cut-off values for nomogram and model-correlated continuous variables.**

| **Variable** | **Cut-off value** | **Youden index** | **Sensitivity** | **Specificity** |
| --- | --- | --- | --- | --- |
| Age, years | 62.5 | 0.192 | 0.536 | 0.781 |
| PNI | 42.2 | 0.238 | 0.825 | 0.603 |
| Nomogram model, point | 197 | 0.319 | 0.853 | 0.781 |

Abbreviation: PNI, prognostic nutrition index.
