# Supplementary material for: Personalized risk prediction for prolonged ileus after minimally invasive colorectal cancer surgery: in-depth risk factor analysis and model development
Source: Int J Colorectal Dis. 2024 Jul 23;39(1):115. doi: 10.1007/s00384-024-04693-w (PMC11266276; doi:10.1007/s00384-024-04693-w)
Supplement: Supplementary file 3 — Supplementary file3 (DOCX 19 KB) [file 384_2024_4693_MOESM3_ESM.docx]

**Table 4**. Comparison of AUCs of nomogram with each independent risk factor

| ***Delong's test*** | | | ***Z* score** | ***P* value** |
| --- | --- | --- | --- | --- |
| Nomogram | *Vs.* | Advanced age | 4.607 | < 0.001 |
| Nomogram | *Vs.* | Male sex | 7.255 | < 0.001 |
| Nomogram | *Vs.* | Age-adjusted CCI score ≥ 6 | 7.399 | < 0.001 |
| Nomogram | *Vs.* | Preoperative PNI | 5.946 | < 0.001 |
| Nomogram | *Vs.* | Preoperative sarcopenia | 8.066 | < 0.001 |
| Nomogram | *Vs.* | Intraoperative fluid overload | 5.635 | < 0.001 |
| Advanced age | *Vs.* | Male sex | 0.344 | 0.730 |
| Advanced age | *Vs.* | Age-adjusted CCI score ≥ 6 | 1.541 | 0.122 |
| Advanced age | *Vs.* | Preoperative PNI | 1.635 | 0.102 |
| Advanced age | *Vs.* | Preoperative sarcopenia | 4.419 | < 0.001 |
| Advanced age | *Vs.* | Intraoperative fluid overload | 4.059 | < 0.001 |
| Male sex | *Vs.* | Age-adjusted CCI score ≥ 6 | 1.876 | 0.061 |
| Male sex | *Vs.* | Preoperative PNI | 1.648 | 0.099 |
| Male sex | *Vs.* | Preoperative sarcopenia | 3.663 | < 0.001 |
| Male sex | *Vs.* | Intraoperative fluid overload | 3.171 | < 0.001 |
| Age-adjusted CCI score ≥ 6 | *Vs.* | Preoperative PNI | 0.068 | 0.946 |
| Age-adjusted CCI score ≥ 6 | *Vs.* | Preoperative sarcopenia | 2.173 | 0.021 |
| Age-adjusted CCI score ≥ 6 | *Vs.* | Intraoperative fluid overload | 1.149 | 0.881 |
| Preoperative PNI | *Vs.* | Preoperative sarcopenia | 2.432 | 0.015 |
| Preoperative PNI | *Vs.* | Intraoperative fluid overload | 1.542 | 0.123 |
| Preoperative sarcopenia | *Vs.* | Intraoperative fluid overload | 0.438 | 0.661 |

Abbreviation: CCI, Charlson Comorbidity Index; PNI, prognostic nutrition index.
